# Association between Exosomal miRNAs and Coronary Artery Disease by Next-Generation Sequencing

**DOI:** 10.3390/cells11010098

**Published:** 2021-12-29

**Authors:** Sheng-Nan Chang, Jien-Jiun Chen, Jo-Hsuan Wu, Yao-Te Chung, Jin-Wun Chen, Chu-Hsuan Chiu, Chia-Ju Liu, Meng-Tsun Liu, Yi-Cheng Chang, Chin Li, Jou-Wei Lin, Juey-Jen Hwang, Wen-Pin Lien

**Affiliations:** 1 Division of Cardiology, Department of Internal Medicine, National Taiwan University Hospital Yunlin Branch, Douliu City 640203, Taiwan; p95421008@ntu.edu.tw (S.-N.C.); jienjiunc@ntu.edu.tw (J.-J.C.); y00703@ms1.ylh.gov.tw (Y.-T.C.); a0988890276@gmail.com (J.-W.C.); drliumt168@hotmail.com (M.-T.L.); jueyhwang@ntu.edu.tw (J.-J.H.); 2 Shiley Eye Institute and Viterbi Family Department of Ophthalmology, University of California, San Diego, CA 92093, USA; uuuuuu1swu@gmail.com; 3 Graduate Institute of Medical Genomics and Proteomics, National Taiwan University, Taipei 100, Taiwan; victor26aaaa@ibms.sinica.edu.tw (C.-H.C.); b83401040@gmail.com (Y.-C.C.); 4 Institute of Biomedical Sciences, Academia Sinica, Taipei 115, Taiwan; 5 Department of Nuclear Medicine, National Taiwan University Hospital, Taipei 100, Taiwan; chiaju.liu@gmail.com; 6 Department of Biomedical Sciences, National Chung-Cheng University, Chia-Yi 62102, Taiwan; biocl@ccu.edu.tw; 7 Department of Internal Medicine, National Taiwan University Hospital, Taipei 100, Taiwan; jeremysnc1000@gmail.com

**Keywords:** coronary artery disease, exosomes, micro RNAs, next-generation sequencing

## Abstract

Background: Among various bio-informative molecules transferred by exosomes between cells, micro RNAs (miRNAs), which remain remarkably stable even after freeze-and-thaw cycles, are excellent candidates for potential biomarkers for coronary artery disease (CAD). Methods: Blood samples were collected from the coronary arteries of 214 patients diagnosed with three-vessel CAD and 140 without CAD. After precipitation extraction, the amounts of exosomes were found to decrease with increased age and three-vessel CAD. Next-generation sequencing was performed to further explore the possible relationship between exosomal miRNAs and CAD. Results: Eight exosomal miRNAs showed altered expression associated with CAD. The up-regulated miRNAs in CAD were miRNA-382-3p, miRNA-432-5p, miRNA-200a-3p, and miRNA-3613-3p. The down-regulated miRNAs were miRNA-125a-5p, miRNA-185-5p, miRNA-151a-3p, and miRNA-328-3p. Conclusion: We successfully demonstrated particular exosomal miRNAs that may serve as future biomarkers for CAD.

## 1. Introduction

Exosomes are actively secreted membrane vesicles that are recognized as the major mediators of intercellular communication [1]. They are 40–100 nm in size and can transfer messenger RNAs (mRNAs), micro RNAs (miRNAs), non-coding RNAs, transcription factors, and proteins between cells [2]. In eukaryotic animals, these extracellular organelles are released into the microenvironment in response to microenvironmental stimuli for cell-to-cell communication [3].

As previously reported, chronic inflammation is involved in the pathogenetic process of atherosclerosis. Some inflammation activities, including proteolysis, calcification, and neovascularization, can lead to the development of atherosclerotic plaques on the arteries, which is diagnosed clinically as coronary artery disease (CAD) [4]. In response to these inflammatory stimuli, the exosomes might be secreted and cause changes within the cardiomyocytes [5].

Among various genetic molecules within the exosomes, miRNAs are one of the most attended and studied elements. miRNAs can remain stable even after freeze-and-thaw cycles, making them excellent candidates for potential biomarkers for CAD [6]. However, little is known about the role of exosomal miRNAs in CAD. With the hypothesis of a possible change in the amounts and contents of exosomes associated with CAD, the current study investigated the altered expression of miRNAs in the pathophysiology of CAD for further clinical application.

## 2. Materials and Methods

### 2.1. Study Population

In this study, 625 patients were enrolled from the National Taiwan University Hospital Yun-Lin Branch, Taiwan, between January 2018 and September 2021. The inclusion criteria were patients hospitalized for elective coronary artery angiography under the impression of stable CAD rather than admitted due to acute coronary syndrome (ACS) [7]. The exclusion criteria were ACS, cardiac arrhythmias, chronic obstructive pulmonary disease, thyroid disease, rheumatic arthritis, malignancy, hepatitis B infection, hepatitis C infection, hemoglobin ≤ 10 g/dL, albumin ≤ 3.2 g/dL, aspartate transaminase ≥ 100 units/L, alanine aminotransferase ≥ 100 units/L, or serum creatinine ≥ 2 mg/dL.

Three-vessel CAD was defined by coronary angiography as ≥ 50% stenosis of the left anterior descendent branch, left circumflex branch, right coronary artery, and/or major branches of each artery in combination with/without the left main coronary artery [8]. The diagnosis of three-vessel CAD was confirmed by coronary angiography, and all the results were interpreted by two cardiologists. The blood samples were collected from the coronary artery during coronary angiography before percutaneous coronary intervention if necessary, and the precipitation method was used for exosome isolation in both participants with/without three-vessel CAD. The amounts of exosomes were calculated by flow cytometry and subsequently verified by Western blot. This study was approved by the Ethics Committee and Institutional Review Board on Human Research of the Medical Research Department of National Taiwan University Hospital, Taipei, Taiwan (201811060RINC/202001100RINB) and adhered to the Declaration of Helsinki. All subjects provided written informed consent before participating in the study. The study workflow is summarized in Supplementary Figure S1.

### 2.2. Blood Samples Collection

Initially, the blood samples were collected in EDTA tubes (10 mL). The blood samples were later centrifugated (3000× *g*, 4 °C, for 10 min) to collect plasma. The plasma was collected in AXYGEN microtubes (MCT-175-C) and stored at −70 °C for further exosome extraction.

### 2.3. Method for Precipitation Extraction

The plasma (2.5 mL) was centrifuged (3000× *g*, 4 °C, for 15 min) to remove cells and cell debris. The supernatant was transferred and filtered with a 0.22 μm membrane (Millipore, Burlington, MA, USA) to a sterile vessel (900 μL). After that, an appropriate volume of exosome precipitation solution (300 μL) (ExoQuick, System Biosciences, Mountain View, CA, USA) was added to the bio-fluid. The sample tubes remained upright and refrigerated (at 4 °C, 30 min). Of note, the tubes should not be rotated or mixed during the incubation period and should remain upright during these steps. The vessels were then centrifuged (1500× *g*, 4 °C, for 30 min). After centrifugation, the exosomes may appear as a beige or white pellet at the bottom of the vessels. The resulting exosome pellet was resuspended in 600 μL sterile 1× PBS with a 0.22 μm membrane filter (Millipore, Merck KGaA, Darmstadt, Germany). The exosome pellets were resuspended in 600 μL sterile 1× PBS for further analysis [9].

### 2.4. Characterization of the Samples of Exosomes

To characterize the samples of exosomes, flow cytometry and Western blot assays were used, and equal protein concentration loading and exosome markers were applied. The particle sizes were assessed with flow cytometry. The standard nanoparticles of 0.1μm, 0.2 μm, 0.5 μm, and 0.8 μm were used to establish the particle size analysis. The Western blot assays were used to detect the exosome markers. Specific markers for exosomes, including CD9, CD63, CD81, and Hsp70, were utilized in this study, and as expected, the standard control markers were found in the exosomes [10].

#### 2.4.1. Flow Cytometric Analysis

The exosomes were filtered with a 0.22 μm membrane (Millipore, Merck KGaA, Darmstadt, Germany) and precipitated. The purified exosomes were suspended in 1× phosphate-buffered saline (PBS). The solution was diluted to 10^4^-fold in the 1× PBS assay for the flow cytometry (CytoFLEX BB10068, Beckman coulter, Brea, CA, USA). The flow cytometry was set to: (1) sample flow rate: 10 μL/mL; (2) events to record: 50,000 events at 0.1μm; (3) time to record: 600 s; (4) volume to record: 10 μL. Because PBS was used for exosome suspension, we used PBS as the reference signal in the flow cytometric analysis [11].

#### 2.4.2. Exosomes Assays by Western Blot

The exosomes were lysed in ice-cold 5× lysis reagent (Promega, Madison, WI, USA). The protein concentration was quantified by Bradford reagent (Sigma, Merck KGaA, Darmstadt, Germany). The 10% sodium dodecyl sulfate polyacrylamide gel electrophoresis (SDS-PAGE) was prepared for protein electrophoresis. Total proteins were mixed with 5× sampling buffer and boiled at 100 °C for 10 min. The mixture and protein markers were loaded in wells for SDS-PAGE at 50 V and 110 V for protein electrophoresis. After protein electrophoresis, separated proteins were transferred to a polyvinylidene difluoride (PVDF) membrane Millipore, Merck KGaA, Darmstadt, Germany) at 95 V for 2 h, and then, 5% skim milk (Sigma, Merck KGaA, Darmstadt, Germany). blocking solution for 1 h at 4 °C with shaking was used for the PVDF membrane. After blocking, the exosome markers were prepared in 1× PBS with 0.05% PBS, 0.1% Tween (PBST) (Sigma, Merck KGaA, Darmstadt, Germany), and 5% skim milk overnight at 4 °C under shaking. We used anti-CD9 antibody (1:1000) (SBI, CA), anti-CD63 antibody (1:20,000) (SBI, CA), anti-CD81 antibody (1:1000) (Abcam, Cambridge, UK), and anti-Hsp70 antibody (1:1000) (SBI, CA) for exosome identification and confirmation. The Bradford method was used for evaluating the quantities of samples for gel electrophoresis. The samples were normalized to the same protein volumes. The catalog numbers and concentrations of the loading proteins were CD9 (5 μg, EXOAB-CD9A-1), CD63 (EXOAB-CD63A-1, 5 μg), CD81 (10 μg, ab109201), and Hsp70 (5 μg, EXOAB-Hsp70A-1) [12].

After primary antibody incubation, membranes with transferred proteins were washed with PBST four times. The secondary antibody (anti-rabbit, 1:10,000) (Millipore, USA) was prepared in PBST with 5% skim milk for 2 h at room temperature under shaking. After the secondary antibody was discarded, the membranes were washed with PBST four times. The enhanced chemiluminescence detection kit (ECL; PerkinElmer, Waltham, MA, USA) was used for identifying the protein bands.

### 2.5. Illumina Next-Generation Sequencing

RNA from purified exosomes was purified using TRIzol reagent. Glycogen was used to facilitate the recovery of small RNA. The specimens were converted to the sequencing library using the Small RNA-Seq Library prep Kit (Lexogen, Vienna, Austria). The manufacturer’ s protocol was closely followed to generate the library. Briefly, purified RNA was ligated to the 10-fold diluted 3′-adapter in the first ligation reaction followed by a column-based purification process to remove excess adaptor. The 5′-adaptor was diluted 10-fold and denatured by heating before ligating to purified small RNA with the 3′-adaptor in the second ligation reaction. Adaptor-ligated RNA was converted to cDNA by reverse transcription and amplified by PCR. The library was further analyzed by non-denaturing polyacrylamide gel and enriched by eluting the desired size DNA library from the gel. The eluted library was subjected to a limited PCR cycle to obtain a sufficient concentration for sequencing in an Illumina NextSeq 550 sequencer for 50 cycles. The sequencing reads were imported to CLC genomic Workbench 21.0.3. Micro RNA was identified by mapping against the miRBase database 22.1. The result was then exported for statistical analysis [13].

### 2.6. Statistical Analyses

Statistical analyses were performed by a researcher who was blinded to the subjects’ conditions. Continuous variables are presented as the mean ± standard deviation (SD) and were compared with a two-tailed Student *t*-test. The categorical variables are reported as frequencies or percentages. The non-normal variables are presented as the median with the interquartile range and were compared with the Mann–Whitney U-test. Multiple linear regression analyses were used to measure the association between the amounts of exosomes and possible co-variables such as age, sex, coronary artery disease, hypertension, and diabetes mellitus. The miRNA expression levels were compared using the Student *t*-test. A two-tailed *p*-value < 0.05 was considered statistically significant. The statistical analyses were performed by the SPSS Version 25.0 statistical software (SPSS, Chicago, IL, USA).

## 3. Results

Among the 625 patients’ post-coronary intervention, 214 patients were diagnosed with three-vessel CAD and categorized into the CAD group. Another 140 patients who were confirmed not to have CAD after coronary angiography were categorized into the patient group. The clinical and laboratory characteristics of these two groups are presented in Table 1. Generally, the mean ages of both groups were over sixty years old, and the patients with three-vessel CAD were older than the patients without CAD (CAD vs. patient: 66.83 ± 10.96 vs. 60.82 ± 11.67; *p* < 0.001) (Table 1). Both groups were predominantly male with more than 50% male participants (CAD: 77%; patient: 69%) (Table 1). In terms of weight, the BMI values were similar between both groups (CAD vs. patient: 26.04 ± 3.93 vs. 26.75 ± 4.00; *p* = 0.1) (Table 1). For the history of systemic diseases, there was a greater percentage of hypertension (HTN) (CAD vs. patient: 70% vs. 54%; *p* = 0.03) and diabetes mellitus (DM) (CAD vs. patient: 46% vs. 26%; *p* = 0.02) diagnosis in the CAD group (Table 1). However, the systolic blood pressure (SBP) (CAD vs. patient: 135.43 ± 26.68 vs. 138.98 ± 24.64; *p* = 0.21) and the hemoglobin A1c (HbA1c) level were not significantly different (CAD vs. patient: 6.47 ± 1.38 vs. 6.38 ± 1.75; *p* = 0.84) (Table 1). The diastolic blood pressure (DBP) was lower in the CAD group (CAD vs. patient: 71.68 ± 14.56 vs. 75.48 ± 13.01; *p*=0.01) (Table 1). The mean creatine level of three-vessel CAD patients was higher (CAD vs. patient: 1.47 ± 1.86 vs. 0.99 ± 0.57; *p* < 0.001), but the appearance of proteinuria did not differ between the two groups (CAD vs. patient: 27% vs. 19%; *p* = 0.13) (Table 1). The value of total cholesterol was higher in the patient group (CAD vs. patient: 155.49 ± 36.23 vs. 166.02 ± 32.66; *p* = 0.01) (Table 1), which might be related to the greater high-density lipoprotein cholesterol level in these patients (CAD vs. patient: 38.4 ± 12.31 vs. 43.58 ± 10.92; *p* = 0.04) (Table 1). Because the pharmacotherapy data were not available in the present study, it could not be verified whether the CAD group was characterized by lower DBP and cholesterol levels due to treatment.

The exosomes were extracted from both groups using the precipitation method for comparison. Interestingly, the amounts of exosomes were greater in the patient group (size ≤ 100 nm, CAD vs. patient: 62,924.22 ± 54,572.57 vs. 76,339.77 ± 48,357; *p* < 0.001) (Table 2). Other sizes of extracellular vesicles more than 100 nm are presented as well to show the distribution of the extracellular vesicles in detail in Table 2. Since patients in the CAD group were older and both groups were predominantly male, we performed multiple linear regression analysis to account for these co-variables. We found that age was an independent factor that correlated negatively with the change in the amounts of exosomes (size ≤ 100 nm; increasing by one year was associated with decreasing 507.29 million particles/mL, *p =* 0.03) (Table 3). However, other factors such as sex, HTN, and DM were not significantly associated with the alteration of exosome amounts (all *p* > 0.05) (Table 3).

After the exosomes were extracted, 52 consecutive samples were further selected from each of the CAD and patient groups for next-generation sequencing (NGS). The basic characteristics of the 104 patients from which the NGS samples were obtained are presented in Supplementary Table S1. When comparing the two subgroups selected for NGS, the mean age of the CAD subgroup was still older than the patient subgroup (CAD vs. patient: 65.04 ± 10.68 vs. 60.65 ± 11.26; *p* = 0.04) (Supplementary Table S1). Both subgroups were predominantly male and had similar profiles for physical appearance, blood pressure, systemic diseases (DM and HTN), and social behavior (Supplementary Table S1). The results of the biochemistry examinations of both subgroups were also within the normal limits (Supplementary Table S1). The amounts of exosomes obtained from both selected groups were also compared, and no significant differences were found after the co-variables were adjusted in the multiple linear regression analysis (Supplementary Tables S2 and S3).

To investigate the possible relationship between stable three-vessel CAD and exosomes, the alteration in exosomal miRNA expression was observed by NGS using samples of the selected subgroups. After comparative exosomal miRNA analysis, eight miRNAs showing up- or down-regulations in stable three-vessel CAD were identified among the 454 exosomal miRNAs examined (Table 4). Among the miRNAs with altered expressions, miRNA-382-3p, miRNA-432-5p, miRNA-200a-3p, and miRNA-3613-3p were noted to be up-regulated in the selected CAD subgroup based on the Mann–Whitney U-test (Table 4). On the contrary, miRNA-125a-5p, miRNA-185-5p, miRNA-151a-3p, and miRNA-328-3p were found to be down-regulated in the selected CAD subgroup (Table 4).

## 4. Discussion

To summarize, there were several important findings and implication of this study: (1) This is by far the largest study utilizing exosomes directly extracted from coronary arteries to investigate the association between exosomes and stable three-vessel CAD. (2) This is also the first study that verifies both stable three-vessel CAD and age as the independent factors associated with changes in the amounts of exosomes. (3) The amounts of exosomes decreased in patients with three-vessel CAD as compared to patients without CAD. (4) The number of exosomes decreased with age, while sex, HTN, and DM did not affect the number of exosomes. (5) We identified altered expression in particular miRNAs that may be associated with the pathogenesis of stable three-vessel CAD. (6) The up-regulated miRNAs in stable three-vessel CAD were miRNA-382-3p, miRNA-432-5p, miRNA-200a-3p, and miRNA-3613-3p. (7) The down-regulated miRNAs in stable three-vessel CAD were miRNA-125a-5p, miRNA-185-5p, miRNA-151a-3p, and miRNA-328-3p.

### 4.1. Variation in the Amounts of Exosomes Associated with CAD and Age

We extracted exosomes directly from the coronary arterial blood and compared the amounts of exosomes between patients with and without CAD using the precipitation method. Strikingly, there were much greater amounts of exosomes obtained from the patient group as compared to the stable three-vessel CAD group (all *p* < 0.05). Even after the covariates were adjusted, stable three-vessel CAD was still negatively associated with the number of exosomes. It has been suggested in past studies that cardiomyocytes may increase the secretion of exosomes in response to stress, such as hypoxia, inflammation, or injury [14]. However, our results showed a decreased number of exosomes in patients with three-vessel CAD, which was different from prior presumptions. Therefore, it is reasonable to suspect that instead of the amount, it may actually be the contents of the exosomes that vary under pathological conditions such as ischemic stimuli. Additionally, we noted that the amounts of exosomes were negatively associated with the aging process, which is a novel finding that has not been reported before (Table 3).

### 4.2. Exosomal miRNAs as the Novel Biomarkers for CAD

miRNAs are endogenous, single-stranded, short, non-coding RNA molecules approximately 22 nt in length. They finetune gene expression by binding to the 3′ untranslated region of the mRNA at a complementary region located approximately at bases 2–8 from the 5′ end, termed the seed region. By matching complementary sequences within the target mRNA molecule, miRNAs could control the expression of functional and biological pathways [15]. Mostly, miRNAs mediate post-transcriptional gene expression by inhibiting mRNA translation or promoting its degradation, resulting in the direct or indirect reduction of protein production. Many biological processes, such as cell proliferation, differentiation, and apoptosis, are reported to be regulated by miRNAs [16]. Thus, miRNAs are considered to play an important role in intercellular communication with relevant roles in various physiological and pathological processes [17].

In comparison with the plasma miRNAs, recent research has revealed that miRNAs within exosomes can remain in a more durable and stable state since the packaged exosomal miRNAs can escape from nuclease degradation under the protection of the lipid bilayer membrane of the exosomes [18]. Moreover, exosomal miRNAs are more sensitive at predicting cardiovascular diseases as compared to circulating plasma miRNAs. For instance, the sensitivity of serum miRNA-208a for ACS diagnosis was inferior to that of exosomal miRNA-208a. The expression of exosomal miRNA-146a was significantly up-regulated in patients with heart failure, and that association was not found from the circulating plasma miRNA-146a. Therefore, exosomal miRNAs have greater potential for investigating cardiovascular diseases than circulating plasma miRNAs [19]. To the best of our knowledge, the current study is the largest study to explore the varied expression of exosomal miRNAs in stable three-vessel CAD by NGS. Most importantly, we successfully demonstrated the up- and down-regulation of particular exosomal miRNAs that may serve as future biomarkers for stable three-vessel CAD.

### 4.3. Association between Exosomal miRNAs and CAD

Our study possesses several strengths as compared to prior studies. In the study conducted by Zhang et al., eight miRNAs known to be involved in the pathogenesis of cardiovascular diseases (miR-192-5p, miR-148b-3p, miR-125a-3p, miR-942-5p, miR-149-5p, miR-32-5p, miR-144- 3p, and miR-142-5p) were selected to investigate the association between exosomal miRNAs and CAD [19]. Since only the eight candidate miRNAs were examined, other previously unidentified exosomal miRNAs associated with CAD were likely missed in their study. In addition, instead of using peripheral blood samples, blood samples collected directly from the coronary arteries were used in this study for exosome extraction. Furthermore, a much greater number of patients were recruited in our work, indicating a better power to detect the relationship between exosomal miRNAs and CAD.

Previously, age and sex were covariates that posed challenges for analysis due to small sample sizes [19,20]. Su et al. used exosomal miRNAs to evaluate the pathophysiological progression from CAD to acute myocardial infraction (AMI) [18]. However, only six AMI and six matching CAD patients were enrolled for miRNA screening, making the age and sex adjustment unavailable in their study [18]. Of note, possible confounders such as age, sex, HTN, and DM were adjusted in the present study to evaluate the association of miRNAs and stable three-vessel CAD, which had not been performed before. However, there might be differences regarding miRNAs between stable CAD, old myocardial infarction, and ACS. Further studies about this issue are needed.

### 4.4. Association among Exosomal miRNAs, Exosome-Originated Protein, and CAD

Notably, Moreira-Costa et al. performed a literature search and network analysis to evaluate the interaction between exosomes and cardiovascular diseases [21]. For CAD patients, up-regulated exosomal proteins, including fibrinogen beta/gamma chain, inter-alpha-trypsin inhibitor heavy chain, and alpha-1 antichymotrypsin, were assessed as putative protein biomarkers. Several down-regulated proteins were also considered relevant to CAD, such as albumin, clusterin, and vitamin-D-binding protein [21]. While our findings provide essential information for the involvement of exosomal miRNAs in stable three-vessel CAD, their results shed light on the possible validation of miRNAs and exosome-originated protein in combination with the novel biomarkers of CAD [21]. Future investigation of exosomal miRNAs in association with relevant proteins may help further elucidate the signaling pathways involved in CAD.

### 4.5. The Up-Regulated Exosomal miRNAs in CAD

Four miRNAs were found with increased expression in there-vessel CAD in this study. Among them, miRNA-382-3p has previously been noted to be associated with the reduction of L-type Ca ^2+^ channel density and pro-arrhythmogenic effects [22]. However, the association between miRNA-382-3p and CAD has not been described. As for miRNA-432-5p, Gao et al. found an increased level of miRNA-432-5p in the peripheral blood mononuclear cells of CAD patients, which was consistent with our findings [23]. The other two miRNAs, miRNA-200a-3p and miRNA-3613-3p, were first identified to be related to the signaling pathways of CAD in our study. Future studies focusing on these miRNAs are needed to verify their association with CAD.

### 4.6. The Down-Regulated Exosomal miRNAs in CAD

We also found four miRNAs that were down-regulated in CAD. miRNA-125a-5p is highly expressed in vascular endothelial cells and plays a role in modulating the inflammatory response within macrophages by repressing a negative regulator of NF-kB signaling pathways [24]. Moreover, it has been reported to modulate lipid uptake and decrease the secretion of inflammatory cytokines in oxidized low-density lipoprotein (oxLDL)-stimulated monocyte-derived macrophages [25]. Therefore, by suppressing oxLDL-induced *endothelin-1* (*ET-1*) expression, miRNA-125a-5p plays a protective role against the development of atherosclerosis [26]. Our findings were consistent with the study by Hao et al., in which miRNA-125a-5p, miRNA-155, and miRNA-199a/b-3p were observed to be down-regulated with the increased expression of *ET-1* and played a crucial role in coronary atherosclerosis [26]. For miRNA-185-5p, it was proven to be involved in the cell proliferation, metastasis, and inflammation of various tumors, but its regulatory role in the progression of CAD remains elusive [25]. As for miRNA-151a-3p and miRNA-328-3p, they were also first reported to be down-regulated in CAD in the current study, and the underlying molecular mechanisms remain to be explored.

In conclusion, this is the first study utilizing exosomes extracted from coronary arterial blood to demonstrate the decreased amounts of exosomes in stable three-vessel CAD patients and aged populations. We also identified eight exosomal miRNAs with altered expression in stable three-vessel CAD. These findings provide novel insights into the molecular pathophysiology of CAD and may serve as the basis for exploring potential biomarkers and predictors of CAD.

## 5. Limitations

In the present study, whether the miRNAs highlighted could be used as biomarkers or are merely associated by chance remains to be conclusively proven. Previously, increased levels of miRNA-432-5p were associated with CAD occurrence, and miRNA-125a-5p had been found to play a protective role against the development of atherosclerosis [23,24,25,26]. Our findings could be regarded as their validation. There were several novel miRNAs that had not been found before in relation to stable CAD in this study. We are preparing another cohort study specifically focused on stable CAD to validate these miRNAs. Further cellular studies about the over-expression or knock-out of these miRNAs can also clarify the signaling pathways of these miRNAs in association with the pathogenesis of stable CAD. Therefore, our findings should be interpreted carefully to avoid false-positive signals. Moreover, there might be differences regarding miRNAs between stable CAD, old MI, and ACS. Further large sample size cohort studies about this issue might clarify this. Finally, this was a cross-sectional cohort study, and we aimed to find the miRNAs connected with severe CAD. Even though we also enrolled patients with stable one-vessel or two-vessel CAD, we did not compare this with patient participants. The miRNAs were analyzed immediately after three-vessel CAD was confirmed by coronary angiography. Therefore, we could not conclude that these miRNAs are related to the pathophysiological progress from one-vessel to three-vessel CAD.

## 6. Conclusions

In summary, this was the first study to use exosomes extracted from blood samples taken from coronary arteries to show that exosomes’ amounts decreased in stable three-vessel CAD patients and during the aging process. We also identified eight exosomal miRNA profiles to serve as potential biomarkers and predictors of stable CAD. These findings provide novel insights into the molecular pathophysiology of CAD.

## Figures and Tables

**Table 1:** Clinical baseline characteristics of the total population.

	CAD (n = 214)	Patient (n = 140)	*p*-Value
	Mean ± SD	Mean ± SD	
Age (y)	66.83 ± 10.96	60.82 ± 11.67	<0.001
Male, N (%)	165 (77%)	96 (69%)	0.08
Waist (cm)	74.72 ± 20.16	82.67 ± 12.29	<0.001
BMI (kg/m^2^)	26.04 ± 3.93	26.75 ± 4.00	0.1
SBP (mm Hg)	135.43 ± 26.68	138.98 ± 24.64	0.21
DBP (mm Hg)	71.68 ± 14.56	75.48 ± 13.01	0.01
HTN	150 (70%)	75 (54%)	0.03
DM	98 (46%)	37 (26%)	0.02
Proteinuria	58 (27%)	27 (19%)	0.13
Smoking	53 (25%)	43 (31%)	0.23
Alcohol	36 (17%)	26 (19%)	0.67
Hb (g/dL)	13.47 ± 2.04	13.95 ± 1.67	0.02
Glu AC (mg/dL)	139.51 ± 56.01	122.67 ± 40.58	<0.001
HbA1C (mg/dL)	6.47 ± 1.38	6.38 ± 1.75	0.84
GOT (U/L)	20.77 ± 6.48	22.06 ± 6.82	0.31
GPT (U/L)	27.61 ± 6.17	29.66 ± 8.08	0.22
BUN (mg/dL)	23.06 ± 14.94	17.43 ± 4.75	<0.001
Cr (mg/dL)	1.47 ± 1.86	0.99 ± 0.57	<0.001
Na (mmol/L)	137.29 ± 2.79	137.6 ± 2.34	0.26
K (mmol/L)	4.05 ± 0.44	4.02 ± 0.36	0.52
CHO (mg/dL)	155.49 ± 36.23	166.02 ± 32.66	0.01
TG (mg/dL)	158.04 ± 104.89	157.8 ± 83.05	0.98
LDL (mg/dL)	91.48 ± 29.24	97.09 ± 27.88	0.08
HDL (mg/dL)	38.4 ± 12.31	43.58 ± 10.92	0.04
CRP (mg/dL)	4.23 ± 6.97	0.13 ± 0.05	<0.001

All results are expressed as means and standard deviations (mean ± SD) for both men and women. CAD, coronary artery disease; BH, body height; BW, body weight; SBP, systolic blood pressure; DBP, diastolic blood pressure; HTN, hypertension; DM, diabetes mellitus; Hb, hemoglobin; Glu AC, fasting blood glucose; HbA1C, hemoglobin A1c; GOT, glutamic oxaloacetic transaminase; GPT, glutamic pyruvic transaminase; BUN, blood urea nitrogen; Cr, creatinine; Na, sodium; K, potassium; CHO, cholesterol; TG, triglyceride; LDL, low-density lipoprotein cholesterol; HDL, high-density lipoprotein cholesterol; CRP, C-reactive protein.

**Table 2:** The amounts of exosomes from the total population.

	CAD (n = 214)			Patient (n = 140)			Mann–Whitney U-Test
	Mean ± SD	Median	IQR	Mean ± SD	Median	IQR	
Size (nm)	Million Particles/mL			Million Particles/mL			*p*-Value
≤100	62,924.22 ± 54,572.57	49,480.92	68,653.44	76,339.77 ± 48,357	67,434.48	65,549.61	<0.001
100~200	9,631.07 ± 10,120.11	6674.68	12,533.43	12,523.47 ± 8767.05	11,719.74	13,367.65	<0.001
≤200	79,914.23 ± 64,822.03	71,042.42	76,675.62	94,487.55 ± 55,614.36	84,257.18	71,795.00	<0.001
200~500	101.33 ± 160.23	34.65	112.62	124.04 ± 148.8	70.06	155.63	0.03

CAD, coronary artery disease; SD, standard deviation; IQR, interquartile range.

**Table 3:** Multiple linear regression for the amounts of exosomes from the total population.

	Size ≤ 100 (nm)		Size ≤ 200 (nm)	
	β (Million Particles/mL)	*p*-value	β (Million Particles/mL)	*p*-Value
CAD	−4218.59	0.02	−5023.20	0.03
Age	−507.29	0.03	−635.71	0.03
Sex	4645.49	0.46	837.41	0.91
HTN	1967.54	0.58	324.58	0.94
DM	3113.07	0.52	5368.82	0.37

CAD, coronary artery disease; HTN, hypertension; DM, diabetes mellitus.

**Table 4:** Comparison of exosomal miRNAs from the selected CAD and patient groups.

miRNA Locus	Selected CAD			Selected Patient			
	N = 46			N = 42			Mann–Whitney U-Test
	Mean ± SD	Median	IQR	Mean ± SD	Median	IQR	*p*-Value
miRNA-382-3p	15.76 ± 35.93	0.00	0.00	3.5 ± 17.17	0.00	0.00	0.02
miRNA-432-5p	11.05 ± 29.4	0.00	0.00	0	0.00	0.00	0.01
miRNA-200a-3p	11.57 ± 31.7	0.00	0.00	0.85 ± 5.48	0.00	0.00	0.03
miRNA-3613-3p	21.96 ± 54.82	0.00	0.00	1.25 ± 8.09	0.00	0.00	0.02
miRNA-125a-5p	765.26 ± 1661.5	0.00	773.54	1945.61 ± 2551.44	242.74	3622.49	0.01
miRNA-185-5p	26.83 ± 50.77	0.00	39.21	55.25 ± 74.74	0.00	92.30	0.05
miRNA-151a-3p	502.46 ± 522.85	339.82	589.45	821.19 ± 745.74	510.97	1030.08	0.02
miRNA-328-3p	2.63 ± 12.8	0.00	0.00	19.5 ± 45.89	0.00	0.00	0.05

CAD, coronary artery disease; miRNA, micro ribonucleic acid; SD, standard deviation; IQR, interquartile range.

## Supplementary Materials

The following supporting information can be downloaded at: https://www.mdpi.com/article/10.3390/cells11010098/s1, Table S1: Clinical baseline characteristics of the selected population for NGS; Table S2: The amounts of exosomes from the selected population for NGS; Table S3: Multiple linear regression for the amounts of exosomes from the selected population for NGS; Figure S1: Study workflow.
