# Supplementary material for: Association between Exosomal miRNAs and Coronary Artery Disease by Next-Generation Sequencing
Source: Cells. 2021 Dec 29;11(1):98. doi: 10.3390/cells11010098 (PMC8750494; doi:10.3390/cells11010098)

cells

MDPI

Supplementary Table S1. Clinical baseline characteristics of the selected population for NGS.

	Selected CAD (n=52)	Selected Patent (n=52)	*p*-value
	Mean ± SD	Mean ± SD	
Age (yrs)	65.04±10.68	60.65±11.26	0.04
Male, N (%)	43 (83%)	34 (65%)	0.04
Waist (cm)	82.65±10.85	82.04±9.24	0.76
BMI (kg/m^2)	26.87±3.80	26.42±3.57	0.53
SBP (mm Hg)	136.56±24.1	138.58±25.36	0.68
DBP (mm Hg)	72.69±10.95	73.67±13.29	0.68
HTN	38 (73%)	32 (62%)	0.14
DM	20 (38%)	14 (27%)	0.21
Proteinuria	13 (25%)	6 (12%)	0.06
Smoking	17 (33%)	23 (44%)	0.23
Alcohol	15 (29%	12 (23%)	0.51
Hb (g/dl)	13.78±1.63	13.82±1.62	0.66
Glu AC (mg/dL)	132.48±33.18	121.75±40.38	0.15
HbA1C (mg/dL)	6.32±0.79	6.8±2.49	0.66
GOT (U/L)	23.64±5.79	19.21±6.17	0.17
GPT (U/L)	26.85±6.01	26.55±7.29	0.82
BUN (mg/dL)	18.84±5.91	17.24±4.6	0.13
Cr (mg/dL)	1.09±0.56	0.92±0.2	0.03
Na (mmol/L)	137.62±1.95	137.58±2.57	0.93
K (mmol/L)	4.07±0.35	4±0.35	0.31
CHO (mg/dL)	151.45±34.61	165.78±29.53	0.03
TG (mg/dL)	149.8±84.49	146.1±66.84	0.81
LDL (mg/dL)	87.96±28.28	99.73±25.11	0.03
HDL (mg/dL)	37.73±5.97	43.88±6.29	0.04
CRP (mg/dL)	0.06±0.01	0.11±0.06	0.35

All results were expressed as means and standard deviations (Mean ± SD) for both men and women. NGS, next-generation sequencing; CAD, coronary artery disease; BH, body height; BW, body weight; SBP, systolic blood pressure; DBP, diastolic blood pressure; HTN, hypertension; DM, diabetes mellitus; Hb, hemoglobin; Glu AC, fasting blood glucose; HbA1C, hemoglobin A1c; GOT, glutamic oxaloacetic transaminase; GPT, glutamic pyruvic transaminase; BUN, blood urea nitrogen; Cr, creatinine; Na, sodium; K, potassium; CHO, cholesterol; TG, triglyceride; LDL, low-density lipoprotein cholesterol; HDL, high-density lipoprotein cholesterol; CRP, C-reactive protein

Supplementary Table S2. The amounts of exosomes from the selected population for NGS.

	Selected CAD (n=52)			Selected Patent (n=52)			Mann–Whitney U test
	Mean ± SD	Median	IQR	Mean ± SD	Median	IQR	
Size (nm)	million particles/ml			million particles/ml			*p*-value
≦100	72410.15±43856.63	74635.72	50913.68	68091.3±32406.54	69982.11	60551.03	0.64
100 ~ 200	12862.35±8429.07	12569.21	11922.17	11948.46±6901.41	11838.52	10203.41	0.65
≦200	86072.63±51906.84	89996.29	63280.61	84371±37143.45	82762.28	72260.87	0.74
200~500	145.48±188.56	72.43	170.80	146.43±170.5	75.08	142.51	0.74

CAD, coronary artery disease; SD, standard deviation; IQR, interquartile-range.

Supplementary Table S3. Multiple linear regression for the amounts of exosomes from the selected population for NGS.

	Size ≦100 (nm)		Size ≦200 (nm)	
	β (million particles/ml)	*p*-value	β (million particles/ml)	*p*-value
CAD	7359.98	0.38	9087.59	0.36
Age	-412.68	0.29	-587.80	0.21
Sex	3350.90	0.72	-1535.56	0.89
HTN	-4280.23	0.27	-5037.36	0.26
DM	4937.89	0.56	5229.06	0.60
NGS, next-generation sequencing; CAD, coronary artery disease; HTN, hypertension; DM, diabetes mellitus				

Supplementary Figure S1. Study workflow.

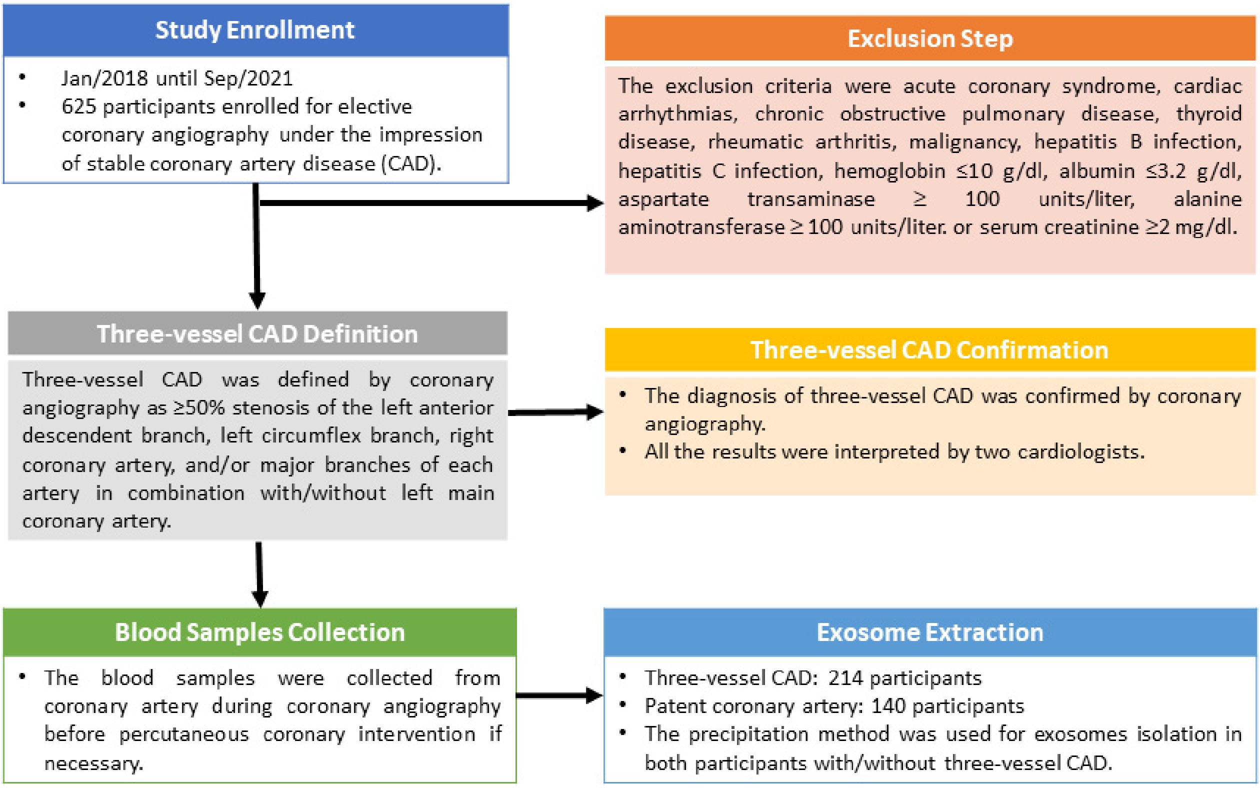

Supplement: Supplementary file 1 [file cells-11-00098-s001.zip › cells-1496939-supplementary.pdf]
